# Enhancing Weigh-in-Motion Systems Accuracy by Considering Camera-Captured Wheel Oscillations

**DOI:** 10.3390/s24248151

**Published:** 2024-12-20

**Authors:** Moritz P. M. Hagmanns, Serge Lamberty, Adrian Fazekas, Markus Oeser

**Affiliations:** 1 Chair and Institute of Highway Engineering, RWTH Aachen University, 52074 Aachen, Germany; 2 Federal Highway Research Institute, 51427 Bergisch Gladbach, Germany

**Keywords:** weigh-in-motion, dynamic load, wheel oscillation, measurement accuracy, data fusion

## Abstract

Weigh-in-motion (WIM) systems aim to estimate a vehicle’s weight by measuring static wheel loads as it passes at highway speed over roadway-embedded sensors. Vehicle oscillations and the resulting dynamic load components are critical factors affecting measurements and limiting accuracy. As of now, a satisfactory solution has yet to be found. This paper discusses a novel correction approach that fuses WIM sensor data with wheel oscillation captured by cameras. In an experiment, a hard plastic speed bump was placed ahead of a piezoelectric WIM sensor to induce oscillation in trucks crossing the WIM sensor. Three high-speed cameras captured the motion of the wheels. The results show that the proposed method improved the accuracy of the measured gross weight for significant wheel oscillations, while no improvement is observed for smaller oscillation amplitudes.

## 1. Introduction

### 1.1. Background and Motivation

Road freight transport plays a fundamental role in modern logistics. Some logistic transport companies overload their trucks in an attempt to reduce their costs. Overloaded trucks cause three significant issues:

1. From a safety perspective, they pose an elevated risk of accidents, with the potential for more severe consequences than trucks that comply with regulations [1];
2. From a structural integrity perspective, overloaded trucks excessively impact road infrastructure wear and tear [1]. To be more precise, the damage of the road is caused by overloaded individual axles as pointed out by Raheel et al. [2];
3. From an environmental perspective, overloaded trucks lead to higher emissions and air pollution. Miftahulkhair et al. [3] showed that for a flexible pavement subjected to long-term overloading of trucks, increased fuel consumption was observed across all vehicles due to the pavement becoming more uneven.

To prevent these negative effects associated with overloaded trucks and, in particular, their axles, enforcement of legal restrictions on axle load and gross weight have been taken. In weight enforcement, one of the strategies deployed by authorities consists of selecting a random sample of trucks for static weighing. Typically, the trucks are not selected objectively but according to the control staff’s experience. Such weighing is conducted on mobile or permanently installed static scales, which require the truck to come to a complete stop for weighing and, therefore, partially disrupt traffic flow. Such static weighing involves the presence of enforcement personnel and the deployment of the scale. For these reasons, this process is labor- and time-intensive, resulting in high costs and only allowing a sample of vehicles to be checked [1].

It is worth developing an accurate system that does not require the presence of enforcement personnel, trucks to exit the main road, or trucks coming to a complete stop for weighing. In this regard, Weigh-In-Motion (WIM) systems are seen as a potential solution [1,4,5]. Sensors within such systems are typically embedded in the pavement surface course. Different sensor technologies utilized for WIM systems exist as reviewed by Dontu et al. [6]. Piezoelectric sensors, bending plates, and load cells are the most commonly utilized sensing technologies. Piezoelectric sensors are in the form of a strip or bar and are only capable of covering a portion of the total footprint of the tire at any given time. In contrast, the other two technologies utilize a wide-width plate sensor that is capable of covering the whole tire footprint. When subjected to a load, all sensor types generate an output signal. This signal is then converted to a mass, which, given a calibration coefficient, provides the equivalent static load. The calibration process requires trucks with different and known loadings and speeds to be driven over the WIM system in order to enable the coefficient fitting.

In the context of weight enforcement, the use of WIM systems is still restricted to the preselection of vehicles as they are currently not accurate enough for direct and automated weight enforcement. A study from Pellizzon et al. [7] from 2022 on WIM systems in Brazil, for example, show that the accuracy varied between 7% to 12% for the vehicle’s gross weight and 15% to 20% for axle group weights depending on the truck type and other factors. With this precision, these systems are especially not as accurate as static weighing systems [6]. The following factors affect the accuracy:

1. Sensor properties (e.g., type [6], installation tilt [8], installation depth [9], and lateral position of crossing [10,11,12]);
2. Environmental aspects (e.g., direction and speed of the wind [8]);
3. Pavement system properties (e.g., road surface evenness [13] and pavement temperature [14]);
4. Vehicle properties (e.g., oscillation [8,15], speed [11,13], tire profile [8,16], and changes in velocity or trajectory [12]).

Amongst these factors, the oscillations of a moving vehicle are often reported to have the most significant impact on accuracy [8,15]. From a conceptual perspective, dynamic weighing with WIM systems significantly differs from static weighing, in which oscillations do not occur. Typical weighing systems, including static weighing and WIM systems, only sense forces (not mass) applied to the pressure-sensitive sensors. The vehicle’s weight is then back-calculated. In the case of a moving vehicle, the WIM system’s detected force is—in general—not equivalent to the gravitational force of the same vehicle at a standstill. Indeed, the unevenness of the road agitates the vehicle, resulting in both horizontal and vertical oscillations, which depend on factors such as the chassis and the driving speed [15]. The vertical oscillations cause a dynamic force component. Therefore, the measuring system is unable to measure the static load directly and can only provide a rough estimate instead.

The correction approach proposed in this article was tested with piezoelectric quartz sensors. Nevertheless, we expect that the correction approach works with different types of sensor technologies as long as it can be assumed that the change in the dynamic load is neglectable while the wheel drives over the sensor (while the sensor measures the dynamic forces). The correction approach works on the physical level of load transfer from the vehicle through the wheels and is in principle independent of the sensor type. This remains to be shown in experiments.

Very few articles deal with the issue of vehicle oscillations in the WIM context, and the associated accuracy issues are not solved yet, resulting in a knowledge gap. This manuscript aims to bridge this gap by enhancing the accuracy of WIM systems through explicit accounting for a vehicle’s oscillation. This research contributes to replacing static weighing systems with WIM systems and, therefore, automating weight enforcement.

### 1.2. State-of-the-Art

Given the identified gap, this literature review focuses on research performed or solutions deployed to consider vehicle oscillations in WIM systems. The most common and almost exclusively followed technique is to increase the number of sensors and average the results. Several studies deal with the accuracy of multiple-sensor WIM (MS-WIM) systems. Below, we review two representative articles as examples.

Cebon and Winkler [16,17] theoretically investigated the design and performance of an MS-WIM array to enhance MS-WIM systems’ accuracy. For this, the authors developed a mathematical model in which a vehicle’s tire exerts a sinusoidal force onto the pavement surface, and an array of evenly spaced idealized noiseless and perfectly accurate sensors was assumed, i.e., the output of each sensor corresponds instantaneously to the exerted force. The output value of the entire array was calculated as the arithmetic mean of all sensors’ determined weights. To assess the accuracy of this array, the authors defined a dimensionless error coefficient. The pavement was considered as a surface whose roughness was described by an arbitrary two-dimensional spectral density. The vehicle was driving at a steady speed. For a vehicle driving at 80 km/h, the model indicated that the error of MS-WIM systems monotonously decreases with an increase in the sensor number until five sensors, after which the error saturates. This error was reduced from 12% with one sensor to 4% with three sensors and 3% with five sensors. The authors validated their model experimentally by deploying 32 tiles with embedded sensors, evenly spaced from each other by 40 cm in the driving direction. Three- and double-axled vehicles drove onto the experimental setup at six different constant speeds, ranging from 8 km/h to 80 km/h, with a total of 460 passes. The authors demonstrated reasonable agreement between practice and theory by feeding the experimental vehicle and pavement setup to the theoretical model. Amongst all passes, utilizing three sensors achieved a Root Mean Squared (RMS) error of 5.7%, and utilizing two sensors had an error of 8.8%. These joint papers display the limitation of utilizing evenly spaced multiple-sensor WIM systems.

Gajda et al. [18] investigated in their study an MS-WIM system that was installed in Poland. It comprises 16 consecutive polymer load sensors evenly spaced at a 1 m distance in the driving direction. The system was divided into eight subsystems, each with two consecutive WIM sensors and one induction loop. The objective of this study was to experimentally and simulatively compare the accuracy of WIM systems equipped with different numbers of evenly spaced sensors. The measurements from each independent sensor were averaged as an arithmetic mean to obtain the final load measurement value. In their experiment, three different preweighed semitrailer trucks passed and drove over the MS-WIM. A total of 36 passes were performed at a constant speed of either 40 km/h, 50 km/h, 60 km/h, or 70 km/h. The accuracy was quantified using the $\delta_{95\%}$ error, which is the relative weighing error that 95% of all observed vehicles fulfill. The results show that the system formed by all 16 sensors reached an error of 8%, while the best-performing subsystem consisting of two sensors yielded even slightly better accuracy, and the worst-performing subsystem had a significant error of 66%. The authors examined how the system accuracy varied with different numbers of subsystems, considering only the best-performing subsystems for each number. The relative error $\delta_{95\%}$ was plotted against the number of sensors, and a second-degree polynomial was fitted. Such a polynomial indicates that an optimal number of sensors with minimal error exists. In this case, eight sensors lead to the best error of approximately 5.5%. The authors also demonstrated a similar behavior numerically, with simulations conducted to assess the accuracy in relation to the number of sensors. Two different cases were distinguished: it was assumed that by adding sensors, each sensor would be 10% or 20% less accurate than the previously added sensor. The result was that both error curves had a minimum and, therefore, an optimal number of sensors. In one additional scenario, the assumption was that all sensors operate with equal uncertainty, and their weighing errors follow a normal distribution with identical parameters. In this case, the error was regressively declining. This indicates that the accuracy gained by additional sensors declines as the number of sensors increases.

Both papers show that even a few sensors can significantly increase the accuracy. However, as the number of sensors in the WIM system increases, so does the cost, and the rate of improvement in measurement accuracy decreases rapidly. Therefore, achieving an accuracy level of a few percent or lower with a reasonable number of sensors is challenging. Finally, it should be noted that taking an average of measured values does not guarantee a certain accuracy.

Given that vehicle oscillations are influenced by uneven road surfaces, an alternative approach is to select a measurement site with an even pavement of superior quality, as proposed by Gajda et al. [19]. The authors furthermore examined the impact of vehicle speed on the dynamic load. To this end, they utilized an instrumented vehicle to measure the dynamic axle load over a 30 m distance. The results indicated that the dynamic wheel load can deviate up to approximately 40% from the static axle load for an 80 km/h vehicle speed. For a vehicle speed of 20 km/h, the weighing error was reduced to a maximum of 10%. We conclude from these results that local speed restrictions at the WIM site can also improve measurement accuracy due to the reduction of vehicle oscillations. While the second approach affects traffic flow, the first approach restricts the choice of locations or requires expensive pavement surface layer renewal. In addition, the evenness of the road can decrease over time.

Zhou et al. [20] proposed an improved evaluation algorithm for load cell WIM systems with the aim of reducing the influence of the dynamic tire forces on the weighing accuracy. The algorithm was based on nonlinear curve fitting of multiple sinusoidal oscillations of different frequencies and a constant that is the static weight. Although the authors demonstrated improvement with simulations and a field experiment, the applicability of the proposed algorithm remains limited. Firstly, the proposed algorithm was based on measuring the total wheel load multiple times during a single rollover. Conventional quartz sensors, common in Europe, can only measure a portion of the whole wheel load at a time due to their small width [6]. The algorithm, therefore, does not apply to this type of sensor. Secondly, the algorithm required low vehicle speeds (the experiment was conducted with a maximum speed of 15 km/h) and excluded high-speed WIM systems designed for highway speeds.

A research project that explicitly accounted for vehicle oscillation is *AKSLast* [9]. The objective was to develop a WIM system for automated weight control. Their approach was to utilize a correction function that explicitly accounted for the unevenness of the road surface and the induced vehicle oscillation. A representative fleet of trucks was selected, and a dynamic wheel load simulation based on road unevenness was conducted using a basic vehicle model. While not clearly stated, we assume that the mean value of the dynamic load of the fleet at the sensor location of this simulation was used to correct the measurement with a constant shift. An experiment demonstrated that this correction approach did not improve the accuracy of the measurement. The authors did not explain this unexpected result. The simulation results indicate a broad range of dynamic loads occurred at the sensor location (±2 kN in 10% to 90% percentile). We assume that a simple constant measurement shift is insufficient for correcting the effects of different truck types and loading conditions.

### 1.3. Objectives and Methodology

This study aims to reduce vehicle oscillation’s impact on the equivalent static load estimation accuracy. This is achieved by designing a correction function utilizing a data fusion approach combining WIM sensor data from only one row of sensors (consisting of two sensors; one per wheel) with wheel oscillation records captured with industrial-grade cameras. For this, raw WIM sensor data from the AKSLast project was used. Within this project, laterally mounted cameras at wheel height additionally collected videos. An optional hard plastic speed bump was installed ahead of the sensors to induce more-than-usual pronounced oscillations. In this scenario, it is anticipated that the dynamics of the load will exert a predominant influence on the accuracy of the measurements. While it allows for the evaluation of the performance of the correction strategy for heavy oscillations, it is not intended to provide a higher level of accuracy than that achieved in the scenario of not using the speed bump and applying the correction function. Therefore, the speed bump is not a component of the correction strategy, but rather a component of its validation. The raw WIM data consisted of voltage signals that were converted to equivalent static wheel loads. Such conversion is basic knowledge (e.g., [21]) and is usually provided by the WIM system manufacturer; therefore, herein, it is not further discussed. In this paper, the wheel deflection is defined as the vertical motion of the wheel center. The deflection of each vehicle’s wheels was tracked using camera recordings. This tracking enabled the comparison of the wheel deflection to a horizontal baseline to understand whether the tire was compressed more than in the steady (static) state or lifted—without necessarily losing contact with the pavement surface. The WIM data and wheel deflection were fused by correlating wheel deflections to equivalent static wheel loads. The correlation was mathematically expressed as a regression, correcting the WIM-obtained dynamic wheel load to estimate the static wheel load. This correction strategy was finally applied for validation to estimate the total gross weight of moving trucks with a known weight.

The structure of this article is organized as follows: Section 2 describes the experimental setup used to gather data. The data fusion approach is presented in Section 3 and explains how individual reference wheel loads were estimated since only the gross weight of each truck was measured with a static scale for reference. Section 4 describes the approach for validating the correction strategy, followed by the combined results and discussion in Section 5. Uncertainties resulting from the experimental setup and data processing are discussed in Section 6. The paper finishes with a conclusion in Section 7.

## 2. Experimental Setup and Data Collection

Within the AKSLast project, the WIM sensors were installed in Germany on the closed and no longer existing section of the A61 highway between the Jackerath and Mönchenglad-bach-Wanlo junctions in the driving direction of Venlo. A row of piezoelectric WIM sensors was installed in the asphalt roadway on the middle lane of three lanes. The sensor row comprised two *Lineas® Quartz Sensor Type 9195GC31* sensors from Kistler Instrumente AG (Winterthur, Switzerland), each covering one rolling track of the trucks’ wheels. The sensors were each 150 cm wide and 5 cm long. An optional speed bump was mounted at a distance of 50 cm, 100 cm, or 150 cm ahead of the sensors to elicit additional truck oscillation. The speed bump, made of hard plastic, consisted of two parts and was 23 cm long, 3 cm high, and 400 cm wide in total. The software used to collect the WIM sensor data was developed by Neurosoft GmbH, Bergisch Gladbach, Germany.

**Figure 1:**
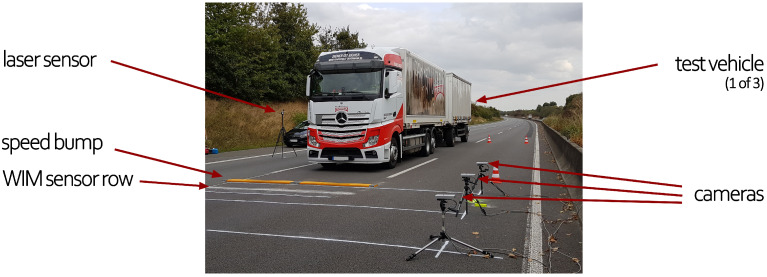
Experimental setup on the German A61 highway used to gather data. A truck is about to cross the speed bump, followed by the WIM sensors. A laser sensor triggers the recordings of the three industrial-grade cameras.

In addition to the WIM sensors, three *Dalsa Genie Nano M2590* cameras were installed on the left lane, recording the movement of the wheels at 60 frames per second. The captured image area was limited due to the high requirements in image resolution. With the sensor pixel dimensions of each camera being 2592 × 2048, we aimed for a resolution of 1 mm/pixel. The cameras were placed and aligned with slightly overlapped images so that approximately 7 m were covered by stitching the images together (2 m ahead of the sensors and 5 m after the sensors). A laser triggered the cameras’ recording as the truck passed by to reduce the amount of image data collected. Figure 1 provides a picture of the above-described experimental setup as a truck is approaching and about to cross the speed bump and sensors.

**Figure 2:**
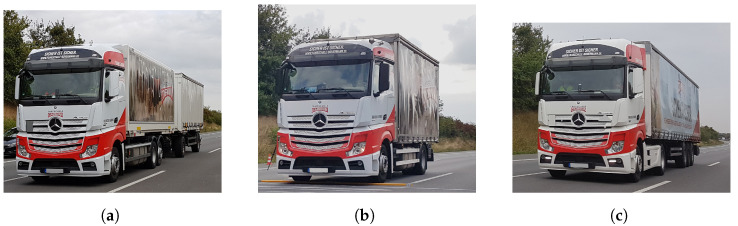
Vehicles utilized for measurement runs: truck with trailer (**a**), truck without trailer (**b**), and semitrailer (**c**).

Three trucks were utilized for the collection of data at the 27th, 28th and 29th of August 2018. Each day, a different truck type was utilized; they are visually displayed in Figure 2 and differed as follows: a 3-axle truck with a 2-axle trailer, a 2-axle truck without a trailer, and a 5-axle semitrailer. The trucks were loaded with several water containers. Three different loading conditions were achieved by varying the filling level, labeled as empty, half-full, and full. The three truck types combined with the three loading conditions resulted in nine different gross weights, determined through static weighing at a nearby gravel pit, where only the static gross weight could be measured.

The trucks drove at a steady speed of 80 km/h, which the driver set in cruise control to maintain a consistent speed. The trucks crossed the experimental setup, turned around at the end of the section, and returned to the starting point on the same section. The bump spacing to the sensor was adjusted after a few measurement runs. Table 1 summarizes the number of passes for each truck type, loading configuration, bump distance, and static gross weight. Additional data were collected during the return trips, considered equivalent to the case without the bump, which are not listed in Table 1.

Ten additional measurement runs were conducted on another day with a specially equipped truck. This vehicle measured the dynamic wheel load in kilograms and the speed with the help of an additional wheel, enabling Neurosoft GmbH to calibrate the WIM system.

## 3. Data Fusion Approach for Correcting the Measurement of Wheel Loads

### 3.1. Estimation of Static Wheel Loads

As mentioned, only the static gross weight of the trucks was measured, suggesting that no reference values were available for the static wheel loads. However, these values are necessary for correcting the effect of the vehicle oscillation, as described in Section 3.3. To estimate reference values of the static wheel loads, we calculated the arithmetic mean of the measured wheel loads from all passes without applying the speed bump and from all return trip passes. We utilized between four and eleven measurement runs for each vehicle and loading condition. The vehicle gross weights resulting from the sum of the estimated static wheel loads deviated by −2.2% (i.e., underestimated) to 3.4% (i.e., overestimated) from the statically measured reference gross weights. Therefore, it is a reasonable assumption that the accuracy of the wheel load estimate is also about this range.

### 3.2. Extraction of Wheel Deflection from Camera Images

**Figure 3:**
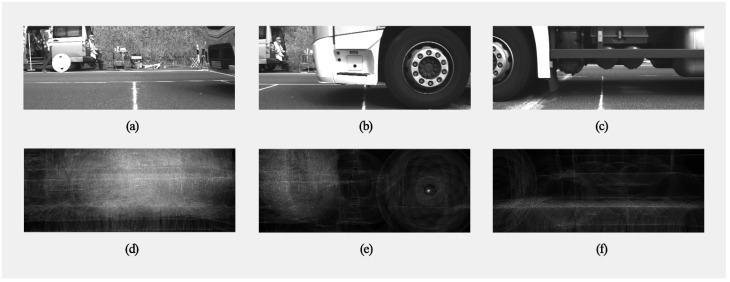
Collage of six example images as a truck passes by. The top row (**a**–**c**) and the bottom row (**d**–**f**) consist of three images taken by the three cameras simultaneously and their Hough-transformed images, respectively. It can be seen, how the center of the wheel creates a white spot in the Hough image (**e**).

The deflection of the wheels was extracted from images recorded by the three cameras. As a preparatory step, the lens distortion was corrected using intrinsic camera parameters derived in laboratory settings. Therefore it was assumed, that the wheels have circular shapes which were identified in the captured images using a (non-real-time) Hough transform algorithm [22]. Since the diameter of the wheels in the image size was unknown, the algorithm was executed for multiple circle diameters. Figure 3 displays a collage of six images as a truck passes by. The top row consists of images taken by the three cameras simultaneously, and the bottom row depicts their corresponding Hough-transformed images. As can be observed, only the middle camera (see Figure 3b) captures the truck wheel in its entirety, whose center is then visible in the Hough transform as a bright point (see Figure 3e). One cannot see any local maxima in the other Hough transforms since no circular shapes can be identified in their corresponding real-world images. The local maxima were extracted from the transformed images. A user performed a manual inspection to confirm that the circular shape was a wheel to remove any false positives.

**Figure 4:**
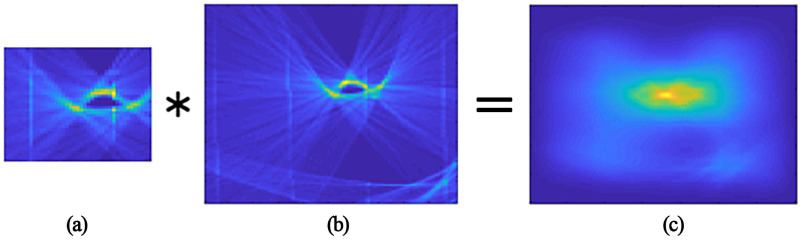
Tracking of the wheel center. The pattern from the Hough transform (**a**) is correlated (operation is denoted with a star symbol *) as a moving window with the timely next consecutive image (**b**), resulting in a heat map indicating where the pattern can be found in the new image (**c**). The local maxima was selected as the new wheel’s center position.

In the following steps, the relative positions of the wheels’ centers in successive images were extracted with respect to the initial detection. Specifically, the wheel centers were tracked by comparing patterns in two successive Hough-transformed images sequentially. To reduce computational effort, a window was cut around the initial pattern from the current iteration (Figure 4a) and a larger search window was cut in the timely subsequent image (Figure 4b), in which the initial pattern is searched. A two-dimensional correlation was then performed between both windows to determine the precise position of the wheel center in the subsequent frame. The correlation results are displayed in Figure 4c as a heat map and show the probability that the initial pattern from the first image matched the pattern from the second image at each position. The local maximum in this heat map, representing the wheel center position with the highest probability, was selected as the most plausible position of the wheel center in the next image. Subsequently, another window was extracted around this position in order to continue tracking the wheel in the same manner until all 16 to 18 frames per wheel pass had been processed.

After tracking the wheels through all three images, the extracted curve was corrected for any potential tilted positioning of the cameras. The pixel values were then transformed into real-world coordinates (mm) using information from the laser distance sensor, which was also used for the triggering mechanism.

### 3.3. Correction of the Wheel Load Measurements

**Figure 5:**
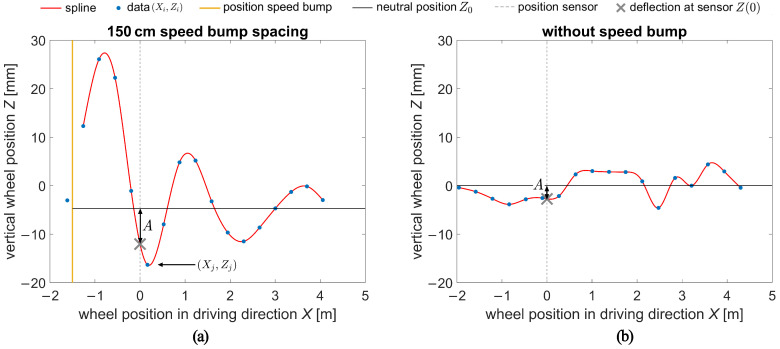
Example for the oscillation of the wheels: 150 cm bump spacing (**a**), without bump (**b**). The deflection A of the wheel at the sensor is depicted visually. The *j* th data point is marked for the 150 cm bump spacing.

To account for the dynamic component in the measurement of a wheel load, it is necessary to know the oscillation state of the wheel at the time of measurement. One possible representation of the wheel load oscillation is the wheel deflection, precisely the vertical motion of the wheel center, denoted herein as Z(X) (mm). *X* (m) represents the longitudinal wheel position in the direction of travel. X=0 m corresponds to the sensor position, and positive values of *X* indicate that the truck wheel has already passed the sensor. Figure 5 graphically represents the wheel deflection as represented by a red line, which is interpolated with a spline between the extracted positions of the wheel’s center points $\left(X_{i},Z_{i}\right)$ for both cases: with and without the bump. A distinct initial peak in the spline can be observed in Figure 5a. On the other hand, in the case without a bump (see Figure 5b), it exhibits erratic oscillatory patterns.

To set the wheel deflection Z(X) in relation to a reference, we define the neutral position $Z_{0}$ as the vertical position of the wheel center that it would have in static equilibrium, i.e., when the vehicle is at a standstill. The *Z*-values resulting from the image processing are interval-scaled, meaning the neutral position is unknown (in general $Z_{0}\neq 0$). Consequently, the neutral position $Z_{0}$ of the wheel oscillation must be estimated. For the determination of $Z_{0}$, the case “with bump” $Z_{0}^{\left(\mathrm{bump}\right)}$ and the case “without bump” $Z_{0}^{\left(\mathrm{without}\;\mathrm{bump}\right)}$ are distinguished since these have different oscillation characteristics as described above. The neutral position $Z_{0}^{\left(\mathrm{bump}\right)}$ is calculated as the arithmetic mean of the wheel deflections $Z_{i}$ starting from and including the first local minimum data point $\left(X_{j},Z_{j}\right)$ of the oscillation, see Figure 5a and Equation (1).

(1) $$Z_{0}^{\left(\mathrm{bump}\right)}=\frac{\sum_{i\geq j}^{n}Z_{i}}{\sum_{i\geq j}^{n}1}$$

where the index *j* corresponds to the first data point after the local minimum and n to the number of data points, which ranges from 16 to 18. This approach was selected as we observed in some cases that the wheels lost contact with the pavement surface immediately after crossing the speed bump so that this problem does not distort $Z_{0}^{\left(\mathrm{bump}\right)}$. More information about this issue is given in Section 4.

The case without a bump showed irregular oscillation behavior (see Figure 5b). That is why the best approach was to estimate the neutral position $Z_{0}^{\left(\mathrm{without}\;\mathrm{bump}\right)}$ as the arithmetic mean of all vertical wheel positions $Z_{i}$ – see Equation (2). Note that the scale of Z(X) was defined during the image extraction process just that the arithmetic mean equals zero. This was due to a missing absolute reference in the vertical direction.

(2) $$Z_{0}^{(\mathrm{without}\;\mathrm{bump})}=\frac{1}{n}\sum_{i=1}^{n}Z_{i}=0$$

As shown in Figure 5a, the wheel position reaches a maximum of almost 33 mm with respect to $Z_{0}$, which is of the order of magnitude of the height of the speed bump of 30 mm.

In our correction strategy, the oscillation state of the wheel at the time of measurement corresponds to the deflection *A* (mm) of the wheel center from its neutral position $Z_{0}$ at the sensor’s location and is calculated according to Equation (3). The sign is defined so that a wheel compression corresponds to a positive deflection *A*.

(3) $$A:=Z_{0}-Z\left(0\right)$$

The vertical wheel position at the sensor Z(0) was interpolated with the spline through 16, 17, or 18 points, corresponding to the number of video frames captured for each truck pass.

**Figure 6:**
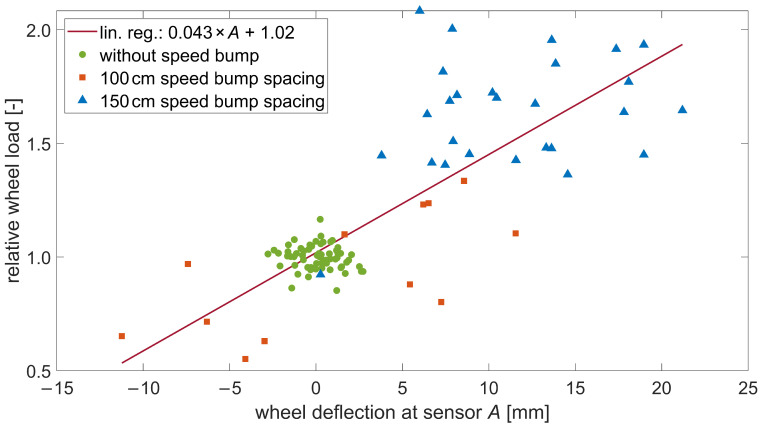
Determination of the correction function (linear regression).

Wheel deflection *A* was related to the dynamic wheel load. To achieve this, the measured wheel loads were converted into relative wheel loads by dividing the measured by the (estimated) static wheel loads. A positive dynamic wheel load component is expected to cause the wheel center to deflect downwards. Wheel deflection *A* should increase with the deviation from the static wheel load. Figure 6 graphically shows the correlation between wheel deflection A and the relative wheel loads. The data has a Pearson correlation coefficient of 0.81, indicating a strong linear relationship. Thus, a linear regression is chosen and fitted as the correction function. The corrected wheel loads $W^{\prime }$ (kg) were obtained by dividing the measured wheel loads *W* (kg) by the correction function as shown in Equation (4). The uncorrected values *W* and corrected values $W^{\prime }$ represent the dynamic and static wheel load, respectively. Due to missing camera data for the right side of the vehicle, the deflections of the right and left wheels are set to be equal: $A_{\mathrm{right}}=A_{\mathrm{left}}=A$. It was implicitly presumed that all wheels of an axle oscillate in the same way. Although this presumption is not in line with expectations of the mechanical properties of vehicle axles, it was necessary due to the limitations of the measurement setup. This leads to uncertainties, as discussed in Section 6.

(4) $$W^{\prime }=\frac{W}{0.043\times A+1.02}$$

## 4. Data Fusion Validation

**Figure 7:**
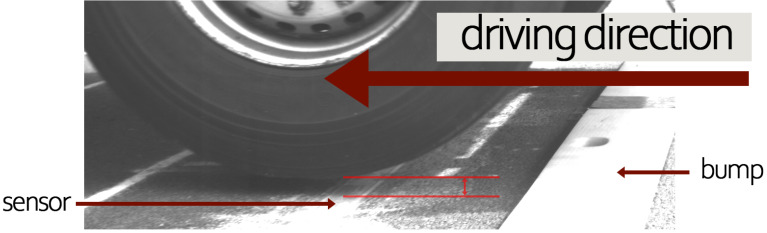
Wheel loses contact with the ground after crossing the speed bump and even misses the sensor.

We applied the data fusion approach and validated it as described in this section. Not all datasets were usable for validation. Upon reviewing the video recordings, it became apparent that the wheels lost contact with the pavement surface immediately after crossing the speed bump, resulting in many wheels not even touching the sensor, an example is shown in Figure 7. In several instances, some truck wheels made contact with the sensors while others did not. It led to incomplete and unusable data sets since we needed all wheels to be observable in the sensor raw data to calculate the total weight for validation. This issue was particularly evident at speed bump distances of 50 cm and 100 cm. We found no (50 cm) and only three (100 cm) complete data sets across all truck types and loading conditions at these bump distances. For reference, for the 150 cm bump distance, we found at least nine complete datasets. Therefore, only the data sets without a bump and spacing of 150 cm were used for validation. The number of data sets used for validation is provided in Table 2.

The primary metric for validation is the gross weight *R* (t) of the trucks, which was measured for each truck and loading condition with a static scale as a reference. In contrast, the real static axle and wheel loads remain unknown, as they have not been subjected to a static weighing process and, therefore, are not included in the validation process. As the counterpart to the reference gross weight *R*, we define the (WIM-measured) total weight *M* (t) as the sum of all WIM-measured wheel loads. Hence, the total weight was not measured directly with the WIM system but calculated from WIM-measured quantities. The total weight *M* was calculated either as the sum of all WIM-measured wheel loads *W* or as the sum of all corrected wheel loads $W^{\prime }$, as defined in Equation (4), which we refer to as the uncorrected and corrected total weight, respectively. The uncorrected and corrected total weights *M* were compared for validation to their static reference gross weights *R*, which were measured in the gravel pit. The quantification of the validation process was based on the concept of trueness and precision [23]. Trueness describes how close the average of the measured values is to the reference value and thus, related to a systematic measurement deviation. Precision describes the scattering of the measured values around their mean value and thus, related to a statistical measurement deviation. Measurements that are both true and precise are accurate. Accuracy refers to the deviation of the measured values from their true values.

As a measure of trueness, bias *B* (t) is defined according to Equation (5).

(5) $$B=\sqrt{\frac{1}{r_{2}-r_{1}}\int_{r_{1}}^{r_{2}}{\left(\mathrm{reg}\left(R\right)-R\right)}^{2}\;dR}$$

R corresponds to the static reference gross weight in tons. The integration range is from $r_{1}=10$ t to $r_{2}=43$ t, as the static gross weights of the reference trucks fall within this range. *A* linear regression reg(*R*) was formed to reflect an average relation between reference weight *R* and measured weight *M*. The bias can be interpreted as a mean systematic deviation over the relevant measurement range. If there were no (systematic) bias, the regression of a large number of measured total weights that are randomly scattered around their static reference gross weight would be congruent with the angle bisector (reg(R)=R), resulting in the measure bias *B* to be zero.

As a measure for precision, the scatter *S* (t) of the measured values around the regression line is defined according to Equation (6).

(6) $$S=\sqrt{\frac{1}{n}\sum_{i=1}^{n}{\left(M_{i}-{\hat{M}}_{i}\right)}^{2}}$$

The variable $M_{i}$ corresponds to the measured total weights, ${\hat{M}}_{i}=\mathrm{reg}\left(R_{i}\right)$ to the regression values, $R_{i}$ to the reference values, and n is the number of data points. It should be noted that the mathematical formula for the scatter *S* is equivalent to the Root Mean Squared Error. However, we have chosen to refer to it as “scatter” to emphasize the meaning of this measure within the concept of trueness and precision. In the absence of statistical deviations, all measured values would lie on the regression line, resulting in zero scatter.

As a measure for accuracy, the (relative) deviation *D* (t) of the measured values from their reference values is defined according to Equation (7).

(7) $$D=\sqrt{\frac{1}{n}\sum_{i=1}^{n}{\left(\frac{M_{i}}{R_{i}}-1\right)}^{2}}$$

The quantity $M_{i}$ corresponds to the measured total weights, the quantity $R_{i}$ to the reference values and n is the number of data points. Ideally, the deviation would also be zero.

## 5. Results and Discussion

**Figure 8:**
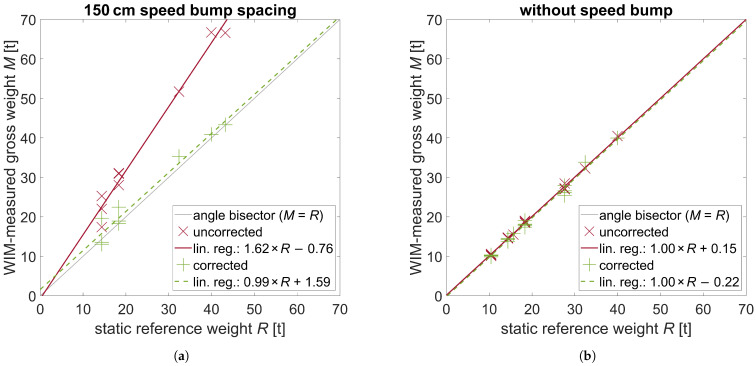
Comparison of the WIM-measured uncorrected and corrected total weights *M* with their reference gross weights *R* for 150 cm bump spacing (**a**) and without bump (**b**).

The uncorrected and corrected WIM-measured total weights *M* were plotted against their reference (static) gross weights *R* in a diagram for comparison. Figure 8a displays the case with the 150 cm speed bump spacing and Figure 8b displays the case without bump. In both charts, uncorrected values are plotted as red crosses (×), while corrected values are plotted as green plus symbols (+). Regression lines were fitted to both the uncorrected (solid red line -) and corrected (dashed green line - - -) data points to show a systematic trend. Ideally, all values should lie on the angle bisector (solid grey line –), i.e., M=R, since the measured total weight would correspond precisely to its reference value. For quantitative analysis, the three measures bias *B*, scatter *S*, and deviation *D* for both the uncorrected and corrected total weights are listed in Table 3.

In the uncorrected data in the case with a speed bump, there is a drastic systematic positive bias in the weights, which can be seen in Figure 8a by the position of the red regression line above the angle bisector. This is because the wheels regain contact with the ground in front of the sensor and the wheel compresses, resulting in a higher dynamic load. Oscillation correction can largely compensate for this effect, resulting in lower deviation *D* and higher accuracy. More precisely, the deviation *D* is reduced by a factor of four, from 60.0% to 15.2% (see Table 3).

Figure 8b shows the case without a bump, with no visual difference between the uncorrected and corrected data and regression lines, which are close to the angle bisector. Table 3 indicates that in the absence of a bump, the corrected measures exhibit a slight increase in bias *B*, scatter *S*, and deviation *D*; although the observed deterioration may not be statistically significant. This behavior is unexpected, because based on the results of the speed bump scenario it could be anticipated that deviation *D* would also decrease by a factor of four, from 2.0% to 0.5%. Such a potential reduction was the objective of the proposed correction approach, but the results show that the value of *D* instead increases to 3.4%. One conclusion might be that in this scenario other influencing factors are more important, dominate the measurement errors, and cannot be reduced with our correction approach. But if this were the case, one would expect that the deviation *D* to decrease by a factor less than four (or even zero), but instead it increased. Therefore, this unexpected behavior is likely due to experimental uncertainty, which will be discussed in the next section.

A comparison of the results of the correction strategy in the presence and absence of the speed bump reveals that the deviation (i.e., the measurement error) of 15.2% in the presence of the speed bump is still approximately an order of magnitude greater than the value of 3.4% observed in its absence. This could mean that, likewise to the scenario without a speed bump, there is too much experimental uncertainty in correction strategy input quantities. Alternatively, it could simply indicate that our approach can correct a large part but not the whole effect of the load dynamics. It is conceivable that a correction function must be established for each vehicle type or even each tire type, or that additional input factors beyond wheel deflection must be considered. Further work with reduced experimental uncertainty and a larger amount of data (vehicle passes and possible input factors) could provide clarity in this regard. It could answer the question which input parameters are essential for the correction methodology.

However, the introduction of a speed bump was not designed to result in a higher level of accuracy than that observed in the case without a speed bump, as the speed bump is not a component of the correction strategy. This result again highlights the significance of an even pavement for achieving high-accuracy measurements, a point previously emphasized by Gajda et al. [19]. In both the corrected and uncorrected scenarios, the accuracy is significantly reduced when the speed bump is employed. The speed bump induces an oscillatory motion like an uneven pavement, although the magnitude is not comparable to that of an ordinary uneven pavement. Nevertheless, it is evident that the dynamic of the loads introduces inaccuracy in the final measurement. The scatter *S* of the measured loads is also greater for the cases in which the speed bump is utilized (see Table 3). This suggests that even in the absence of any measurement bias, which was introduced in the experiments by the speed bump and may not be representative for uneven pavement, the accuracy is influenced negatively through higher statistical fluctuation of the measurements caused by the stronger influence of the load dynamics. However, load oscillations will occur even with a high-quality road surface, resulting in unavoidable inaccuracies. These should be eliminated in the future through the application of our proposed correction approach. The objective is to enhance the results of our correction approach, e.g., by reducing experimental uncertainties, which will be discussed in the following section.

The main purpose of this fundamental research is to gain insights into the relationship between wheel oscillation and the dynamic vehicle load and thus, a possible correction of the measurements to increase the accuracy. It is not the intention to solve all issues for the implementation in a real system. However, given that the ultimate goal is to utilize this knowledge in commercial devices, it is still worthwhile to briefly discuss the challenges, limitations, and potential of the technology employed. The proposed correction strategy is based on the fusion of data from WIM sensors with that from cameras. The cameras must fulfill industrial standards to withstand harsh environmental conditions like extreme temperature spread, humidity, sunlight, and mechanical impacts throughout the year. Optical cameras are constrained by visibility conditions. For example, operations at night, in rain, fog, or snow can be challenging or impossible. A remedy could be the usage of thermal cameras that provide reliable images even under such conditions. These would have to be equipped with an automatic cleaning system to cope with the expected contamination from dust and dirt. Another challenge could be the obscuring of the target vehicle by other vehicles in lanes between the camera and the target vehicle. The automated weight enforcement targets overloaded trucks. Therefore, a possible solution to this issue could be the introduction of an overtaking ban for trucks at the WIM site. This would require trucks to drive on the outer lane, thereby ensuring an unobstructed view from at least one side. Future work is needed to assess whether this is sufficient. If it is not sufficient and cameras have to be placed on both sides, the system would be unable to cover 100% of the trucks. In general, it must be considered that such a system may not be able to cover 100% of all vehicles and conditions. However, in the opinion of the authors, this would still offer a significant improvement compared to the current situation (random checks) and is also accepted in the context of speed monitoring, for example. Despite all challenges the fusion of cameras with WIM sensor data might enhance, on the other hand, vehicle classification and traffic monitoring, which are supplementary functions that a WIM system can potentially perform. For instance, computer vision algorithms can be utilized to count vehicles or axles, or maybe even to detect specific truck types directly, thereby supporting the aforementioned functions. However, this is not the immediate objective of our present research, and it remains to be addressed in future work.

## 6. Method Uncertainties Affecting Correction Results

Although we demonstrated the potential of this novel correction strategy for significant wheel oscillations, we could not prove it for minor wheel oscillations in the absence of a bump, which is relevant for real-world applications. When considering only the acquired data without a bump (green and round data points in Figure 6), a negligible Pearson correlation coefficient of −0.15 was obtained. The linear correction function used to describe this relationship is insufficient. This lack of correlation is likely due to the following uncertainties:

- Vertical wheel position measurement. Indeed, this primarily depends on the camera’s resolution and secondarily, the corresponding Hough transform algorithm. The cameras were set to achieve a resolution of 1 mm/pixel, taking the lateral position of the trucks into account. Therefore, we can assume that the uncertainty in the vertical position of the wheel is at least 1 pixel or 1 mm, respectively. Figure 5b shows that the span of the vertical wheel motion in the instance without a speed bump is about 10 mm. Therefore, 1 mm corresponds to 10% uncertainty in this case;
- Neutral position $Z_{0}$. Because of the complex oscillation form and the limited captured segment, the accuracy of the estimation of the neutral position $Z_{0}$ was limited. A misestimation of 1 mm also leads to at least 10% uncertainty in the case without the speed bump shown in Figure 5b;
- Wheel deflection *A*. This is a combination of the two first uncertainties. *A* is about 3 mm in the example of Figure 5b. If the combination of the two above-mentioned uncertainties leads, for example, to a miscalculation of 1 mm, this yields in this example an error of 33% concerning *A*;
- Right-hand side vertical wheel movement. Because of technical limitations and the high complexity of the experimental setup, only the wheel oscillation on the left side of the trucks could be captured and we had to use it for correction on both sides;
- Static reference wheel loads. We had to use estimated static reference wheel loads instead of measured ones. We estimated the uncertainty at least about 3% (see Section 3.1);
- Additional influencing factors. Finally, it is possible that the vertical wheel movement alone cannot accurately represent the wheel load oscillation due to additional influencing factors that were not considered in this study. Factors like tire type or pressure or yet unknown factors might affect the correction strategy’s outcome. This effect might be observable, especially in the small wheel oscillation amplitudes that occurred in the case without a speed bump.

The combined effect of these uncertainties had likely obscured any improvement in the results without a speed bump. It might also explain why the corrected measurements for the bump case are still a magnitude worse than the measurements without a bump. To reduce uncertainty in future experiments and address the mentioned points, we recommend the following changes to the experimental setup:

- Use a higher-resolution camera or higher-focal-length lens to cover the wheels as large as possible and reduce the mm per pixel ratio;
- Record wheel deflection over a longer distance using more cameras to increase the accuracy of the estimate of the neutral position $Z_{0}$ by averaging more values;
- Record wheel deflection on both sides of the trucks using cameras;
- Measure individual static wheel loads as a reference using static weighing;
- Record more parameters that might influence the wheel load oscillation, such as tire type and pressure;
- Perform more test runs to increase the statistical validity;
- Place the speed bump at least 2 m in front of the sensor(s).

## 7. Conclusions

This paper presents a correction strategy to reduce the effect of vehicle oscillation on WIM measurements. The methodology was based on a fusion of WIM sensor data with wheel oscillation camera recordings. It is one of the few approaches in the scientific community that explicitly accounts for the oscillation of the wheels or wheel load, respectively. Our approach has the advantage that only a single row of WIM sensors is required. We showed that this approach works for intense wheel oscillation but found no improvement for minor wheel oscillation.

The WIM sensor data and camera recordings were gathered within the AKSLast project. It consisted of wheel loads, measured with two piezoelectric WIM sensors, one for each wheel. Several truck types (see Figure 2) with different loading conditions were utilized for passing over the WIM sensors at different speeds. An optional speed bump (see Figure 1) was installed to induce heavy oscillation of the trucks so that the dynamics of the load was the dominant influencing factor in these scenarios. This allowed us to evaluate the performance of the proposed correction approach in the validation process. The movement of the wheels was obtained with three overlapping camera images using a Hough transform algorithm (see Figure 3 and Figure 4). We correlated the wheel deflection (see Figure 5) and the relative wheel load to obtain a correction function (see Figure 6), which was subsequently validated by estimating the gross weight of trucks. For the validation, the corrected total weight was calculated as the sum of all corrected wheel loads. This was compared to the reference gross weight of the trucks, measured separately in a gravel pit. The comparison indicates that the data fusion correction strategy drastically enhances the accuracy of (static) gross weight measurement for the case with the speed bump. The error decreased from 60% to 15% (see Table 3). The result further demonstrates the significance of maintaining an even pavement surface for precise measurements. In the practically relevant case without a bump, experimental uncertainties (e.g., determining the vertical position of the wheel) likely obscured any improvement in the results. Uncertainty in the input variables of the correction strategy might also explain the remaining error of 15% in the scenario in which the speed bump is present. But it could also indicate that the correction methodology needs to be improved to further eliminate the effect of load dynamics. Only future work with reduced experimental uncertainty and a larger amount of data could provide clarity in this regard. Therefore, in Section 6 we have discussed sources of uncertainty and recommendations to reduce them in future experiments. At the end of the discussion in Section 5, we also briefly discussed the challenges, limitations, and potential of using cameras at WIM sites.

For future work, in view of the most common solution to increase accuracy, an interesting approach is applying the data fusion strategy to several consecutive WIM sensor rows (MS-WIM). Such a setup enables the comparison of load oscillation obtained via WIM sensors and wheel oscillation captured with cameras. This allows the narrowing down of other plausible influencing factors and provides valuable insights as a research approach. This strategy is an explicit way to deal with the influence of vehicle oscillation and will ultimately allow automated weight enforcement, likely reducing the number of overweight trucks on the roads.

## Figures and Tables

**Table 1:** Number of passes for each truck type, loading condition, and bump distance. The static gross weight of trucks is indicated as a reference.

	Truck with Trailer			Truck Without Trailer			Semitrailer		
**Speed Bump**	**Empty**	**Half-Full**	**Full**	**Empty**	**Half-Full**	**Full**	**Empty**	**Half-Full**	**Full**
without	1	2	3	5	5	5	3	5	3
50 cm	1	1	1	3	3	3	1	1	1
100 cm	1	1	1	3	3	3	1	1	1
150 cm	1	2	1	3	3	3	1	1	1
gross weigth [kg]	21,310	32,400	43,220	10,420	14,360	18,340	15,640	27,620	39,960

**Table 2:** Number of data sets evaluated, as differentiated by truck type, loading condition, and bump distance.

	Truck with Trailer			Truck Without Trailer			Semitrailer			
**Bump**	**Empty**	**Half-Full**	**Full**	**Empty**	**Half-Full**	**Full**	**Empty**	**Half-Full**	**Full**	**Total**
without	0	1	0	5	5	5	1	4	1	22
150 cm	0	1	1	0	3	3	0	0	1	9

**Table 3:** Determined measurements for the WIM-measured total static weight *M*.

	150 cm Speed Bump Spcaing			Without Speed Bump		
	**Bias B**	**Scatter S**	**Deviation D**	**Bias B**	**Scatter S**	**Deviation D**
	**[kg]**	**[kg]**	**[%]**	**[kg]**	**[kg]**	**[%]**
uncorrected	16,801	2536	60.0	97	376	2.0
corrected	1273	2145	15.2	308	725	3.4

## Data Availability

Dataset available on request from the authors.
